# Device Engineering for All-Inorganic Perovskite Light-Emitting Diodes

**DOI:** 10.3390/nano9071007

**Published:** 2019-07-12

**Authors:** Dongxiang Luo, Qizan Chen, Ying Qiu, Menglong Zhang, Baiquan Liu

**Affiliations:** 1 School of Materials and Energy, Guangdong University of Technology, Guangzhou 510006, China; 2 Guangdong R&D Center for Technological Economy, Guangzhou 510000, China; 3 Institute of Semiconductors, South China Normal University, Guangzhou 510000, China; 4 State Key Laboratory of Luminescent Materials and Devices, South China University of Technology, Guangzhou 510640, China; 5 LUMINOUS! Centre of Excellent for Semiconductor Lighting and Displays, School of Electrical and Electronic Engineering, Nanyang Technological University, Nanyang Avenue, Singapore 639798, Singapore

**Keywords:** light-emitting diode, all-inorganic perovskite, charge injection, charge balance, charge leakage

## Abstract

Recently, all-inorganic perovskite light-emitting diodes (PeLEDs) have attracted both academic and industrial interest thanks to their outstanding properties, such as high efficiency, bright luminance, excellent color purity, low cost and potentially good operational stability. Apart from the design and treatment of all-inorganic emitters, the device engineering is another significant factor to guarantee the high performance. In this review, we have summarized the state-of-the-art concepts for device engineering in all-inorganic PeLEDs, where the charge injection, transport, balance and leakage play a critical role in the performance. First, we have described the fundamental concepts of all-inorganic PeLEDs. Then, we have introduced the enhancement of device engineering in all-inorganic PeLEDs. Particularly, we have comprehensively highlighted the emergence of all-inorganic PeLEDs, strategies to improve the hole injection, approaches to enhance the electron injection, schemes to increase the charge balance and methods to decrease the charge leakage. Finally, we have clarified the issues and ways to further enhance the performance of all-inorganic PeLEDs.

## 1. Introduction

In the recent years, halide perovskites have emerged as a novel class of optoelectronic materials for many fields (e.g., solar cells, lasers and photodetectors) due to their excellent properties including size-tunable optical bandgaps, narrow emission and excellent charge-transport capabilities [1,2,3,4,5,6]. In particular, the property of size-tunable optical bandgaps in perovskites is the same as that of conventional metal chalcogenide semiconductors [7]. For example, Protesescu et al. demonstrated size-dependent photoluminescence (PL) emission from CsPbBr_3_ nanocrystals, where the PL peak shifted from 512 to 460 nm with the edge size decreasing from 11.8 to 3.8 nm [8]. Additionally, the perovskite sizes can be controlled by reaction temperatures to tune the bandgap (e.g., the size decreases by decreasing the reaction temperature) [9]. Such outstanding characteristics also render that halide perovskites can be used as the emitters for light-emitting diodes (LEDs) [10,11,12,13,14,15]. In 2014, Tan et al. reported the first hybrid organic–inorganic CH_3_NH_3_PbBr_3_ perovskite LED (PeLED) [16]. Although the external quantum efficiency (EQE) of their green device is only 0.1%, this groundbreaking finding unlocks a new door for the application of perovskites in LEDs. Since then, PeLEDs have rapidly attracted a great deal of attention from both academic and industrial researchers. To date, some groups have realized PeLEDs with EQE exceeding 20% [17,18].

In 2015, Zeng et al. demonstrated all-inorganic PeLEDs, where CsPb(Cl/Br)_3_, CsPbBr_3_ and CsPb(Br/I)_3_ were used as the blue, green and orange emitter, respectively [19]. This finding showed that all-inorganic perovskites could be a new class of emitters in LEDs, despite the EQE of the devices is low (e.g., an EQE of 0.12% for the green all-inorganic PeLEDs). Compared with hybrid organic–inorganic perovskite materials, all-inorganic perovskites (e.g., CsPbX_3_, X = I, Br and Cl or mixed halide systems Br/I and Cl/Br) have better thermal stability [20,21,22], which is more beneficial to the practical use. In addition, all-inorganic perovskites could possess excellent PL quantum yield (PLQY, e.g., near unity in solution), ensuring the development of highly efficient all-inorganic PeLEDs [23,24,25,26]. Furthermore, all-inorganic perovskites can show exceptional color purity (e.g., full width at half-maximum (FWHM) <20 nm) and be compatible with the solution-processable technique, triggering intense interest in applying them for PeLEDs [27,28,29,30]. Therefore, since the first report of all-inorganic PeLED [19], the performances of all-inorganic PeLEDs (e.g., EQE, current efficiency, power efficiency, luminance or brightness, lifetime and voltage) have been greatly enhanced over the past five years [31,32,33,34,35]. Currently, the highest EQE of all-inorganic PeLEDs is above 20% [36], which is comparable to the state-of-the-art organic LEDs (OLEDs) [37,38,39,40,41] and CdSe-based quantum-dot LEDs (QD-LEDs) [42,43,44,45,46]. As a matter of fact, a large number of high-efficiency all-inorganic perovskite LEDs have been reported. Before achieving the record EQE of 21.3% for red devices [36], Kido’s group reported highly efficient green all-inorganic perovskite LEDs. For example, an EQE of 8.73% was obtained via effective washing process and interfacial energy level alignment, which was the best efficiency in 2017 [47]. They also obtained a green all-inorganic perovskite LED with an EQE of 8.08% by exploiting low-dielectric-constant washing solvent “diglyme” [48]. After the first report of all-inorganic PeLEDs, Zeng’s group proposed a series of strategies to improve the device performance. By controlling the ligand density, they achieved a green PeLEDs with an EQE of 6.27% [49]. Later, an EQE of 11.7% was attained through room-temperature triple-ligand surface engineering [50] and an EQE of 16.48% was yielded via organic–inorganic hybrid passivation [51]. For red all-inorganic PeLEDs, Yu’s group designed a plenty of methods to increase the efficiency. An EQE of 6.3% was reported by enhancing the electron injection [52], an EQE of 11.8% was realized with the utilization of PbS capped CsPbI_3_ emitter [53] and an EQE of 8.2% was achieved for flexible red devices [54]. In the case of blue all-inorganic PeLEDs, the record EQE is 1.9% [55]. Thus, the all-inorganic PeLED technology is believed to be promising for the future-generation displays and lighting [56,57,58,59,60].

To develop high-performance all-inorganic PeLEDs, one crucial way is the optimization of emitting materials CsPbX_3_ [61,62,63,64,65,66]. In fact, most of the reports about all-inorganic PeLEDs are mainly focused on this way. For the further development of all-inorganic PeLEDs, the innovation of device engineering is essential and may be more critical [67,68,69,70,71]. For example, Rogach et al. sandwiched a thin film of perfluorinated ionomer (PFI) between the perovskite emissive layer (EML) and hole transport layer (HTL) to improve the hole injection, resulting in three times enhancement for the peak luminance of CsPbBr_3_ PeLEDs [72]. To improve the device engineering, one scheme is to explore the unique characteristic of all-inorganic PeLEDs. In addition, by drawing on the reported concepts in OLEDs, CdSe-based QD-LEDs and other related optoelectronic technologies [73,74,75,76,77], high-performance all-inorganic PeLEDs can be expected via the enhancement of device engineering.

Herein, the state-of-the-art concepts for device engineering in all-inorganic PeLEDs will be summarized, where the charge injection, transport, balance and leakage have a great influence on the performance. First, the fundamental concepts of all-inorganic PeLEDs will be described. Then, the enhancement of device engineering in all-inorganic PeLEDs will be introduced. At last, the issues and ways to further enhance the performance of all-inorganic PeLEDs will be briefly clarified.

## 2. Fundamental Concepts of All-Inorganic PeLEDs

### 2.1. All-Inorganic Perovskite Emitters

**Figure 1:**
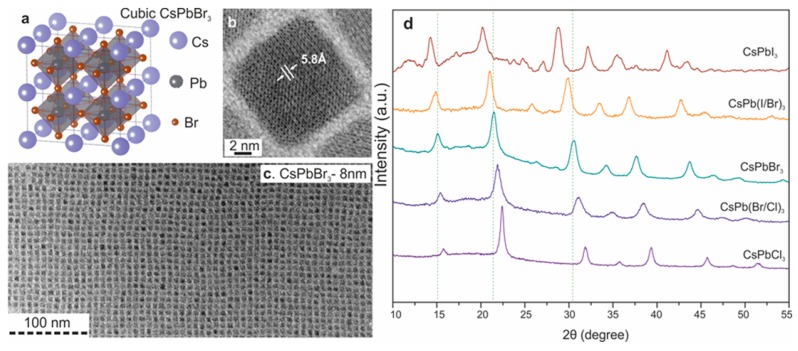
Monodisperse CsPbX_3_ nanocrystals. (**a**) Schematic of the cubic perovskite lattice; (**b**,**c**) transmission electron microscopy (TEM) images of CsPbBr_3_ nanocrystals; (**d**) X-ray diffraction patterns for typical ternary and mixed-halide nanocrystals. Reproduced from Reference [8].

CsPbX_3_ is isostructural to perovskite CaTiO_3_ and related oxides, which has been studied for more than 60 years [78]. In 2015, Kovalenko et al. for the first time reported the successful form of colloidal CsPbX_3_ nanocrystal QDs (4–15 nm edge lengths), which showed visible spectral region of 410–700 nm, narrow PL emission FWHM of 12–42 nm, wide color gamut, high PLQYs of up to 90%, and short radiative lifetimes in the range of 1–29 ns [8]. Figure 1 depicts the typical monodisperse CsPbX_3_ nanocrystal QDs synthesized via the hot-injection method and their structural characterization. However, it is noted that the hot-injection method may exhibit some shortcomings (e.g., high temperature, inert atmosphere and high localization of injected agents exist in the hot-injection procedures). To loosen this bottleneck, Zeng et al. in 2016 reported a facile and high-yield fabrication of stable CsPbX_3_ QDs with high optical merits, where the PLQY could be up to 95% via supersaturated recrystallization at room temperature, free from inert-gas protection and injection operation [79]. For CsPbX_3_ nanocrystals, their optical characteristics can be tuned by adjusting the composition of halide ions, varying the degree of the cations, and controlling the size of perovskite nanocrystals because of the quantum confinement effect [80]. With such tunable properties, CsPbX_3_ nanocrystals have vast potential to be one of the excellent emitters [81,82,83,84,85]. In PeLEDs, the ligand density of CsPbX_3_ nanocrystals has an important influence on the performance. This is because ligands have double-side effect: (i) Enough ligands can give surface passivation to eliminate surface defects, ensuring high PLQY and ink stability; (ii) excessive ligands form insulating layers as oleylamine and oleic acid organics show weak electric conductivity, preventing the charge injection [86,87,88,89,90,91,92]. Therefore, how to achieve the trade-off between surface passivation and charge injection via ligands control is key to all-inorganic PeLEDs.

Aside from the colloidal nanocrystals, CsPbX_3_ thin films, which are achieved by spin-coating or vacuum-evaporating the precursors, can be functioned the emitters of all-inorganic PeLEDs. In 2005, Yantara et al. reported the first all-inorganic PeLED based on CsPbBr_3_ thin films through controlled modulation of the trap density by varying the CsBr-PbBr_2_ precursor concentration, achieving a maximum luminance of 407 cd m^−2^ [93]. In the case of vacuum-evaporating CsPbX_3_ thin films, Liao et al. demonstrated efficient all-inorganic PeLEDs in 2017 via co-evaporation of CsBr and PbBr_2_ based on a vacuum thermal evaporation process, yielding a maximum EQE of 1.55% [94]. Thus, the various strategies of forming CsPbX_3_ emitters have inspired remarkable interest in the investigation of electrically driven devices [95,96,97,98,99].

### 2.2. Device Architectures

**Figure 2:**
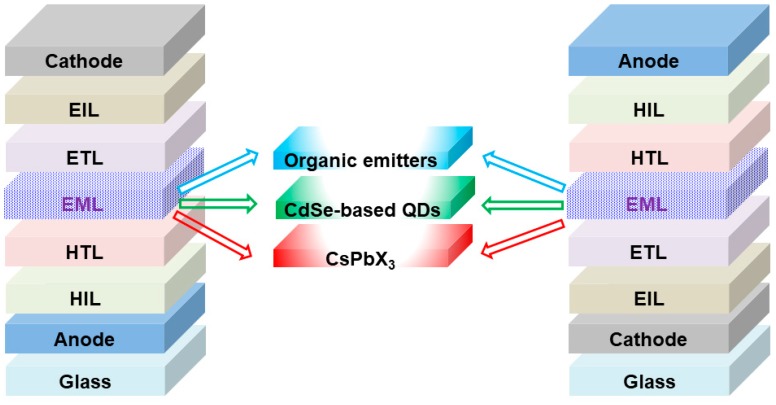
The diagram of device architectures of LEDs with various emitters, i.e., organic emitters for organic LEDs (OLEDs), CdSe-based quantum dots (QDs) and CsPbX_3_ for all-inorganic perovskite LEDs (PeLEDs). Left: The normal structures. Right: The inverted structures.

Similar to OLEDs and CdSe-based QD-LEDs, all-inorganic PeLEDs can be realized via the normal or inverted architectures [100,101,102,103,104], as shown in Figure 2. For the electroluminescent processes, holes and electrons will be first injected from anode and cathode, respectively. Then, by virtue of the hole injection layer (HIL) and HTL, holes will reach the EML. On the other hand, electrons will arrive at the EML via the electron injection layer (EIL) and electron transport layer (ETL). With the insertion of all-inorganic perovskite emitters between HTL and ETL, holes and electrons will meet each other to form excitons for radiative recombination. As a result, emissions will be generated [105,106,107,108,109]. Due to the almost similar device architectures, many concepts in OLEDs and CdSe-based QD-LEDs can be applied into all-inorganic PeLEDs. This may be also the reason for the fact that the EQE of all-inorganic PeLEDs can quickly overtake 20% in about five years.

Although both normal and inverted device architectures can be utilized to build all-inorganic PeLEDs, high-performance devices are generally constructed by the normal architectures. As a matter of fact, most of all-inorganic PeLEDs are reported with normal architectures [110,111,112,113,114,115]. One of the reasons is that the energy levels of all-inorganic perovskite are well matched with neighboring charge transport layers, which ensures enough charges can be reached the EML. For example, the valence band maximum (VBM) of CsPbBr_3_ is 5.9 eV [116], matching the highest occupied molecular orbital (HOMO) of typical HTLs, including poly-(*N*-vinylcarbazole) (PVK, 5.6 eV) and poly(*N*,*N*′-bis(4-butylphenyl-*N*,*N*′-bis(phenyl) benzidine) (poly-TPD, 5.4 eV) [116]. On the other hand, the conduction band minimum (CBM) of CsPbBr_3_ is 3.6 eV [105], which is much lower than the lowest unoccupied molecular orbital (LUMO) of representative 1,3,5-tris(Nphenyl-benzimidazol-2-yl)benzene (TPBi, 2.7 eV) ETL [117], indicating that the electrons are barrier-free when they are transported from the ETL to the EML [118,119,120,121,122]. Therefore, both holes and electrons can be readily transported within the normal architectures. However, it is deserved to point out that the ETL, EIL and cathode of all-inorganic PeLEDs with normal architectures are usually deposited by the vacuum-evaporated technology instead of solution-processed technique. The negligible attention was paid on all-solution-processed devices may be attributed to the fact the penetration effect between ETL and perovskites is still challenging to resolve, although the all-solution-processed technique exhibits more merits (e.g., lower cost, simpler fabrication and less time) [123,124,125,126,127].

For the inverted architectures, the most widely used EIL or ETL is zinc oxide (ZnO) due to its high electron mobility (~1.3 × 10^−3^ cm^2^ V^−1^ s^−1^) [87]. However, there is an electron barrier between the CBM of ZnO (4.4 eV) and CsPbBr_3_ (3.6 eV), although the electron barrier between the CBM of ZnO and the work function of indium tin oxide (ITO, ~4.7 eV) is small [128,129,130]. Hence, it is not easy for the electron injection in inverted all-inorganic PeLEDs. By adopting more efficient ETLs, modifying the typical ZnO ETL, or inserting an interlayer between the ETL and EML to reduce the interfacial barrier [131,132,133,134,135], high-performance devices may be expected. Considering the fact that the most efficient PeLEDs were realized by using normal device architectures [17,18], there is much room for the further investigation of inverted architecture.

## 3. The Device Engineering in All-Inorganic PeLEDs

### 3.1. Basic Aspects of Device Engineering

According to the above concepts, aside from the consideration of perovskite emitters, the innovation of device engineering is another element to determine the performance of all-inorganic PeLEDs. After the selection of perovskites, the EQE is vitally affected by the charge balance from the perspective of device engineering [136,137,138,139,140,141,142,143,144,145]. This is because the EQE in LEDs is defined as follows [146,147,148,149,150]:

(1) $$\mathrm{EQE}=\eta_{out}\cdot r\cdot q\cdot \gamma ,$$

where *η_out_* represents the outcoupling factor, *r* represents the fraction of excitons that potentially radiatively decay, *q* represents the PLQY and *γ* represents the charge balance. In general, *η_out_* is not influenced by the internal operation of LEDs, while *r* and *q* are fixed by emitters. Thus, *γ* plays a significant role in the device efficiency. In fact, how to manage the charge injection, transport, balance and leakage are key to achieve satisfactory performances, i.e., high efficiency, bright luminance and long lifetime [151,152,153,154,155]. In addition, the power efficiency is inversely proportional to the operational voltage [156,157,158,159,160]. Hence, the low voltage is essential to produce high power efficiency.

To enhance the device engineering, enough holes and electrons are required to meet at the perovskite EMLs, forming excitons (i.e., excited electron−hole pairs [96]) for emissions. Generally, the large exciton binding energy and the short exciton diffusion length are required to enhance the efficiency of PeLEDs [161]. Thus, the charge injection and transport should be very effectively manipulated [162,163,164]. In addition, the number of holes is ideal to be equal to that of electrons, guaranteeing the charge balance [165,166,167]. Moreover, to reduce the charge leakage, uniform EML films and charge blocking layers are desirable [168,169,170,171,172,173]. As a matter of fact, some innovations of device engineering in all-inorganic PeLEDs were reported based on these concepts, which will be introduced in the following sections.

### 3.2. Emergence of All-Inorganic PeLEDs

**Figure 3:**
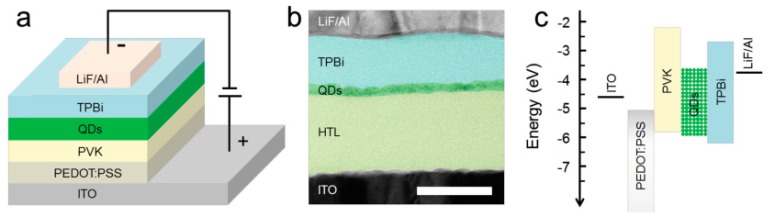
Illustration of PeLEDs. (**a**) The device structure. (**b**) Cross-sectional TEM image showing the multiple layers. Scale bar, 50 nm. (**c**) Flat-band energy level diagram. Reproduced from reference [19], with permission from John Wiley and Sons, 2015.

At the initial stage for the development of halide perovskite optoelectronic devices, most of the attentions were paid on solar cells. With the step-by-step discovery of amazing characteristics of perovskite materials, researchers are motivated to fabricate hybrid organic-inorganic PeLEDs. To solve the stability issue of organic-inorganic perovskites, all-inorganic PeLEDs are developed [174,175,176,177,178]. In 2015, Zeng et al. took the first step to construct all-inorganic PeLEDs, where the emitters CsPbX_3_ (X = Cl, Br, I) QDs were synthesized through hot-injecting cesium stearate (CsSt) to PbBr_2_ solution [19]. As shown in Figure 3, the device architecture was ITO/poly(ethylenedioxythiophene):polystyrene sulfonate (PEDOT:PSS, 40 nm), PVK (10 nm)/perovskite QDs (10 nm)/TPBi (40 nm)/LiF (1 nm)/Al (100 nm), in which QDs were CsPb(Cl/Br)_3_, CsPbBr_3_ and CsPb(Br/I)_3_ for blue, green and orange electroluminescence, respectively. For the blue, green, and orange PeLEDs, luminances of 742, 946 and 528 cd m^−2^, with EQEs of 0.07%, 0.12% and 0.09% were achieved, respectively. In their device architecture, TPBi was employed as ETL, while PVK was used as HTL and electron blocking layers. PVK could reduce the hole injection barrier, block the electron in the active layer, and hence allow holes and electrons to recombine in EMLs. As a result, no any notable parasitic emissions originated from the charge transport layer could be observed in the entire electroluminescence spectrum under varied voltages. In fact, such device architecture is very effective and becomes one of the most popular architectures. For example, the most efficient blue all-inorganic PeLED was fabricated via this architecture, yielding the EQE of 1.9% [55].

**Figure 4:**
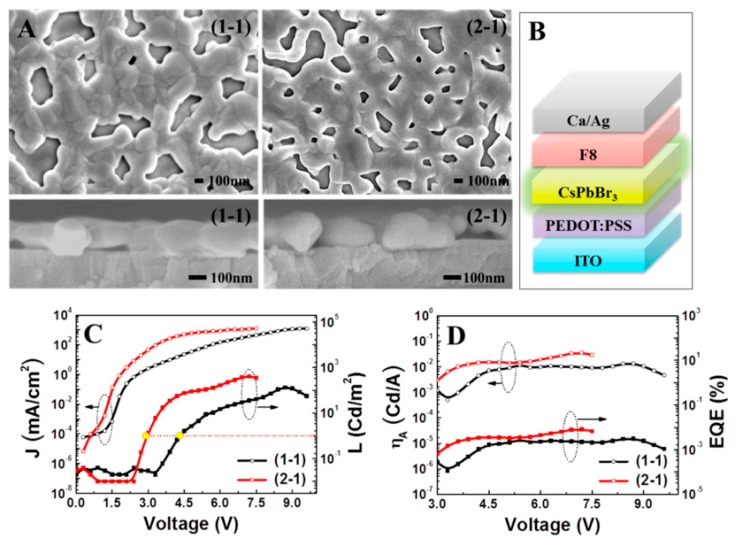
Topographic and cross-sectional images of (1-1) and (2-1) samples (**A**) together with the generic schematic diagram of the devices (**B**). Current density and luminescence versus driving voltage curves (**C**) and current efficiency (η_A_) and external quantum efficiency (EQE) versus voltage curves (**D**) of all samples. Reproduced from reference [93], with permission from American Chemical Society, 2015.

At the almost same time, Yantara et al. reported all-inorganic PeLEDs by using CsPbBr_3_ thin films as the emitter [93]. As shown in Figure 4, the device architecture was ITO/PEDOT:PSS/CsPbBr_3_/poly(9,9-di-n-octylfluorenyl-2,7-diyl) (F8)/Ca (20 nm)/Al (80 nm), in which the trap density of low temperature (70 °C) solution-processed CsPbBr_3_ was reduced by varying their precursor concentration (i.e., using CsBr-rich solution). For varied CsBr/PbBr_2_ ratio (i.e., 0.8, 1.0, 2.0 and 3.0), the outcome of the reactions in DMSO solvent was different. Clear solutions were obtained from CsBr/PbBr_2_ ratio ≤1.0 (after stirring for 5 h in room temperature), while precipitates were observed for CsBr/PbBr_2_ ratio ≥2.0 (i.e., CsBr-rich solutions). Polycrystalline CsPbBr_3_ film with additional CsPb_2_Br_5_ phase was observed on the film deposited from the solution with CsBr/PbBr_2_ ratio of 0.8. The CsPb_2_Br_5_ peak was disappeared with increasing the CsBr/PbBr_2_ ratio and pure CsPbBr_3_ were observed for films with CsBr/PbBr_2_ ratio ≥1.0. Due to the lower trap density, the maximum luminance of the PeLED using the 2.0 CsBr/PbBr_2_ solution (named 2-1, 407 cd m^−2^) was higher than that of the PeLED utilizing the equimolar CsBr/PbBr_2_ solution (named 1-1, 132 cd m^−2^). For their device architecture, F8 was acted as an EIL, which was dissolved in chlorobenzene (10 mg/mL) and spun onto the CsPbBr_3_ EML. Hence, the device could be called all solution-processed PeLED [179,180,181,182,183]. However, the devices suffered from a mismatch in the injection barrier of holes and electrons, since approximately 2 V discrepancy existed between the current turn-on voltage and the turn-on voltage for the electroluminescence.

### 3.3. Strategies to Improve the Hole Injection

Owing to the multilayer device architectures, charge barriers easily exist between the adjacent layers [184,185,186,187,188]. As a consequence, charges are not smoothly transported to the EML in all-inorganic PeLEDs, reducing the probability of exciton generation. In addition, charges can be accumulated at the interface of charge transport layers and EML, leading to the nonradiative recombination [189,190,191,192]. Therefore, it is expected that high-performance all-inorganic PeLEDs can be organized via the enhancement of charge injection and transport. In particular, since there is usually a large barrier for the hole transport in all-inorganic PeLEDs, how to effectively improve the hole injection and transport is crucial to the performance. So far, three main strategies have been reported to improve the hole injection and transport, (i) the insertion of an interlayer at the interface of the neighboring layers, forming a step-wise layer for the hole transport; (ii) the control of the energy level alignment by modifying the HILs or HTLs; (iii) the use of materials with high hole mobilities [193,194,195,196,197,198]. With such strategies, suppressed charging perovskites, prevented exciton quenching together with excellent film morphology may be simultaneously obtained, further enhancing the performance of all-inorganic PeLEDs.

**Figure 5:**
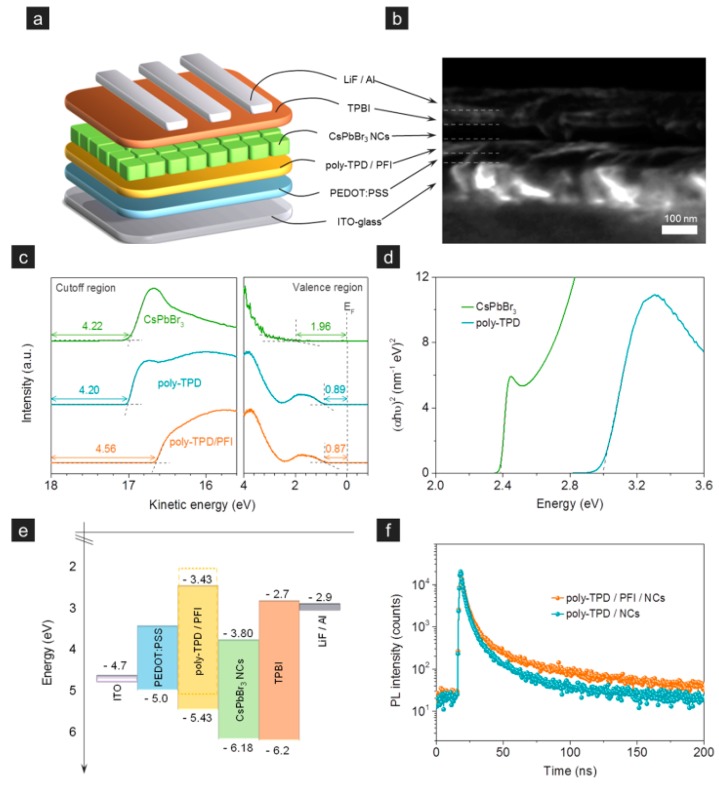
(**a**) Device structure and (**b**) cross-sectional SEM image of the PeLED. (**c**) Ultraviolet photoelectron spectroscopy (UPS) spectra of CsPbBr_3_, poly-TPD and poly-TPD/PFI film deposited on ITO. (**d**) Tauc plot of CsPbBr_3_ and poly-TPD films on quartz substrates. (**e**) Energy band diagram. (**f**) Photoluminescence (PL) decay curves of a CsPbBr_3_ film on a poly-TPD, and as a film on a PFI/poly-TPD. Reproduced from reference [72], with permission from American Chemical Society, 2016.

For the first strategy, Rogach et al. improved the hole injection by incorporating ∼5 nm PFI between the HTL and perovskite EML, leading to a 0.34 eV increase of the VBM of HTL [72]. As shown in Figure 5, the device architecture was ITO/PEDOT:PSS (25 nm)/poly-TPD (40 nm)/CsPbBr_3_ nanocrystal film (40 nm)/TPBI (40 nm)/LiF/Al. With the use of thin PFI, the VBM of poly-TPD HTL was increased, which was beneficial to the hole injection, ensuring efficient radiative recombination of excitons in the EML. Besides, PFI suppressed charging CsPbBr_3_ emitters, which were formed when CsPbBr_3_ was directly contacted with poly-TPD since a spontaneous charge transfer process occurred due to the large energy level difference. As a consequence, a three-fold increase in peak brightness reaching 1377 cd m^−2^ was achieved via the insertion of PFI. From the same way, Lee et al. inserted PFI between PEDOT:PSS and CH_3_NH_3_PbBr_3_ in the hybrid organic-inorganic PeLEDs, achieving the highest current efficiency of 15.5 cd A^−1^ at that time [199]. In their devices, PFI could facilitate the hole injection, prevent exciton quenching at the PEDOT:PSS/perovskite particle film interface, and induce uniform perovskite films possibly due to the low surface energy (∼23 mN/m) [200].

**Figure 6:**
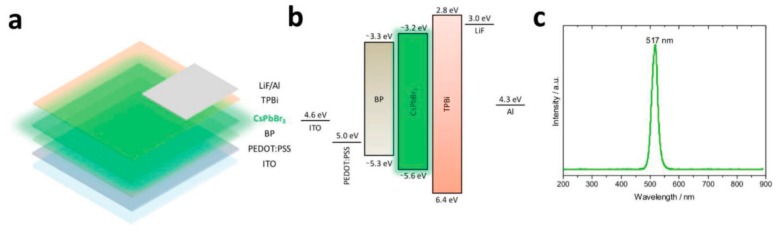
(**a**) Schematic illustration of the device architecture and (**b**) the energy band alignment diagram. (**c**) EL spectrum at 6 V. Reproduced from reference [201], with permission from John Wiley and Sons, 2018.

The introduction of PFI or metal oxides can enhance the performance of PeLEDs, however, such materials may form aggregates and yield to wettability problems with the subsequent deposited perovskites, resulting in electrical shorts and power losses. To alleviate this difficulty, Blom et al. inserted the 2D material black phosphorus (BP) between PEDOT:PSS and CsPbBr_3_ as a HTL to reduce the injection barrier [201]. BP had a HOMO of 5.32 eV, which lowered the injection barrier by 0.3 eV. As shown in Figure 6, the device architecture was ITO/PEDOT:PSS/BP/polycrystalline CsPbBr_3_/TPBi/LiF/Al, where solution-processable, large and ultrathin BP flakes with fewer defects were synthesized by electrochemical exfoliation of bulk BP. By virtue of BP, the EQE was enhanced to 2.8%, which was four-fold higher than that of control device without BP (0.07%). The enhanced performance was attributed to the fact that BP decreased the hole injection barrier and gave rise to uniform growth of the perovskite, resulting in a higher injected hole current and lower leakage current and nonradiative losses, respectively.

**Figure 7:**
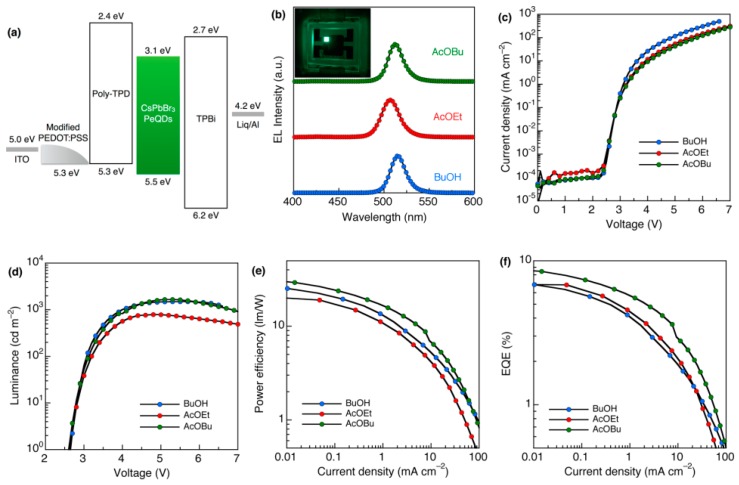
PeLEDs performance. (**a**) Energy diagram. (**b**) EL spectra of a device at 25 mA cm^−2^. Inset: Emission image of the device. (**c**) Current density–voltage characteristics, (**d**) luminance–voltage characteristics, (**e**) power efficiency–current density characteristics and (**f**) EQE–current density characteristics. Reproduced from reference [47], with permission from American Chemical Society, 2017.

PEDOT:PSS is a well-known HIL in solution-processed LEDs due to its optical and electrical properties, such as smoothing the surface of substrates, reducing the current leakage and facilitating the hole injection [202,203,204]. However, there is still a hole barrier between PEDOT:PSS and HTLs, reducing the hole transport. To overcome this issue, Kido et al. modified PEDOT:PSS with nafion to greatly enhance the work function from 4.72 to 5.27 eV, which was close to the HOMO of the poly-TPD (5.31 eV) [47]. Hence, the hole injection barrier between modified PEDOT:PSS and poly-TPD is quite small. As shown in Figure 7, the device architecture was ITO (130 nm)/modified PEDOT:PSS (40 nm)/poly-TPD (20 nm)/CsPbBr_3_/TPBi (50 nm)/lithium 8-quinolate (Liq, 1 nm)/Al (100 nm), where the excess ligands of CsPbBr_3_ QDs were washed by butanol (BuOH), hexane and ethylacetate (AcOEt) and butylacetate (AcOBu). With such effective device architecture by controlling the energy level alignment, the PeLED based on CsPbBr_3_ QDs washed with AcOBu exhibited a maximum power efficiency of 31.7 lm W^−1^ and EQE of 8.73%, which was the highest among all-inorganic PeLEDs at that time. Later, Kido et al. also used the modified PEDOT:PSS with nafion to develop PeLEDs, achieving an EQE of 8.08% for the device using CsPbBr_3_ with a short ligand didodecyl dimethyl ammonium bromide (DDAB) [48]. Thus, the control of the energy level alignment of HILs or HTLs is an effective strategy to enhance the performance.

**Figure 8:**
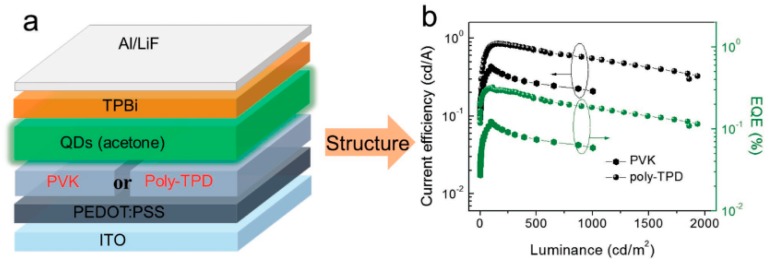
Contributions from selection of hole transport layer (HTL). (**a**) Schematic illustration of device for structure optimization and (**b**) corresponding comparison of current efficiency and EQE. Reproduced from reference [49], with permission from John Wiley and Sons, 2016.

The use of charge transport materials with high mobility is another effective strategy to boost the charge injection and transport, which has been broadly exploited in various types of LEDs [205,206,207,208,209,210]. In the case of all-inorganic PeLEDs, Zeng et al. replaced the PVK HTL with the poly-TPD HTL, achieving a 2.6-fold higher EQE [49]. As shown in Figure 8, the device architecture was ITO/PEDOT:PSS (30 nm)/HTL (40 nm)/CsPbBr_3_/TPBi (40 nm)/LiF (1 nm)/Al (100 nm), where the HTL was PVK or poly-TPD. The enhancement of EQE for the poly-TPD based device could be attributed to the fact that the hole mobility of poly-TPD (~1 × 10^−4^ cm^2^ V^−1^ s^−1^) is two orders of magnitude higher than that of PVK (~1 × 10^−6^ cm^2^ V^−1^ s^−1^), which improved the hole injection. With the selection of more effective solvents for the treatment of CsPbBr_3_ QDs (appropriate hexane/ethyl acetate treating cycles), the EQE was enhanced to 6.27%. Furthermore, Kido et al. used the almost similar device architecture (i.e., ITO/PEDOT:PSS/poly-TPD/perovskites/TPBi/Liq/Al) with efficient red emitter CsPb(Br/I)_3_, realizing the first all-inorganic PeLED with EQE of >20% [36].

### 3.4. Approaches to Enhance the Electron Injection

As mentioned above, the electron transport between the organic ETLs and the EML is usually barrier-free for all-inorganic PeLEDs with normal device architectures. In terms of inorganic ETLs (e.g., the most commonly used ZnO), the electron injection efficiency should be considered due to the barrier existing between the ETL and perovskite EMLs [211]. To enhance the electron injection, some approaches were proposed, including (i) the exploitation of impurity doped inorganic ETLs to optimize the energy band alignment, (ii) the employment of interfacial engineering between inorganic ETLs and perovskite EMLs.

**Figure 9:**
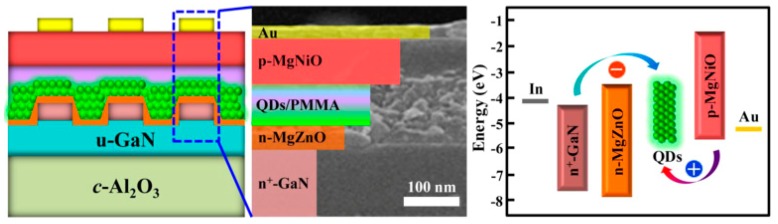
Schematic diagram, cross-sectional SEM image and energy band alignment of the PeLEDs. Reproduced from reference [212], with permission from American Chemical Society, 2017.

For the first approach, Shi et al. enhanced the electron injection through energy band engineering of carrier injectors by Mg incorporation and the thickness optimization, attaining an inverted all-inorganic PeLED with the maximum EQE of 2.39% [212]. As shown in Figure 9, the device architecture was double-polished c-Al_2_O_3_ substrates/patterned low-resistance n^+^-GaN (2 μm)/n-Mg_0.38_Zn_0.62_O (45 nm)/CsPbBr_3_ QDs (55 nm)/p-Mg_0.23_Ni_0.77_O (80 nm)/Au (30 nm), in which n^+^-GaN was used as the electron source, conducting template and a transparent window. Due to the matched electron affinity with CsPbBr_3_ and a deep VBM, n-type MgZnO was deemed as the electron-injection and hole-blocking layer. Specifically, the decreased electron affinity from −3.6 eV (ZnO) to −3.25 eV (Mg_0.38_Zn_0.62_O) favored an almost barrier-free electron injection process. Besides, the VBM above −7.2 eV for Mg_0.38_Zn_0.62_O implied a favorable hole-blocking effect and thereby provided a leakage suppression. Analogously, an Mg^2+^ content of ∼23% (mole percent) was alloyed in the NiO film to adjust its conduction and valence bands for a matched energy level with CsPbBr_3_, guaranteeing the hole injection. Thus, an efficient and stable PeLED was developed. Owing to MgZnO possessing effective electron injection and hole confining ability, Shi et al. then used it to prepare all-inorganic PeLEDs with the normal architecture of ITO/p-NiO/CsPbBr_3_ QDs/Mg_0.2_Zn_0.8_O/Al, achieving an EQE of 3.79% [213]. Furthermore, Tan et al. for the first time used the solution-processed ZnMgO as the ETL in a normal device architecture of ITO/NiOx/CsPbBr_3_/ZnMgO/Al, obtaining a maximum luminance of 17017 cd m^−2^ and a current efficiency of 3.41 cd A^−1^ [214]. Additionally, lithium-doped TiO_2_ has also been demonstrated to be an excellent ETL in all-inorganic PeLEDs [215,216].

For the enhancement of interfacial engineering, a thin multifunctional layer (e.g., polyethyleneimine (PEI), polyethylenimine ethoxylated (PEIE)) is generally introduced between inorganic ETLs and EMLs [217,218]. With such interfacial layer, not only the perovskite film formation quality during solution processing can be enhanced, but also the work function of cathode contacts is lowered. Huang et al. for the first time investigated this approach in hybrid organic-inorganic PeLEDs with the device architecture of ITO/PEI-modified ZnO (20 nm)/CH_3_NH_3_PbI_3-x_Cl_x_ (50 nm)/poly(9,9-dioctyl-fluorene-co-*N*-(4-butylphenyl)diphenylamine) (TFB, 25 nm)/molybdenum oxide (MoO_x_, 8 nm)/Au (100 nm) [219].

**Figure 10:**
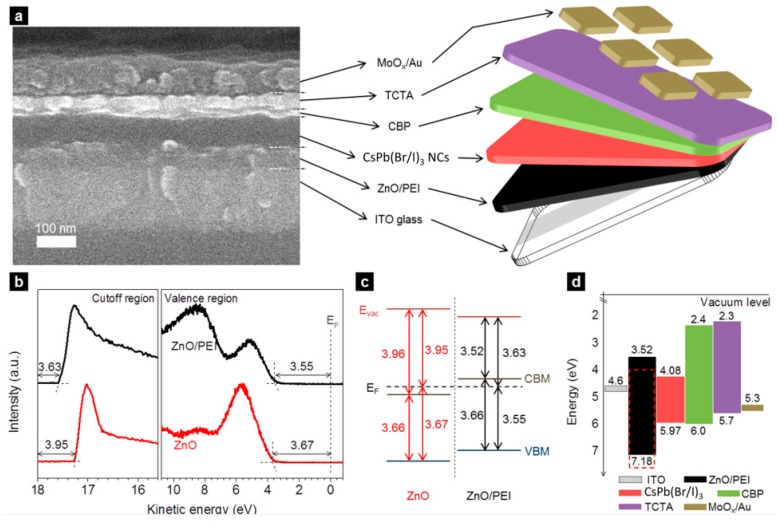
Structure and energy levels. (**a**) Device structure and cross-sectional SEM image of a PeLED. (**b**) UPS spectra of ZnO and ZnO/PEI film deposited on ITO glass substrate. (**c**) Energy level diagram. (**d**) Overall energy band diagram of the LED structure. The red dash line represents for energy levels of ZnO. Reproduced from reference [52], with permission from American Chemical Society, 2016.

In the case of all-inorganic PeLEDs, Yu et al. also demonstrated that the use of PEI between the ZnO ETL and the EML was effective to realize high-efficiency devices, since PEI facilitated the electron injection and prevented the charging of emitters [52]. After the PEI modification, a reduction of 0.44 eV in the CBM of small ZnO nanocrystals (~2 nm) was achieved, providing an efficient electron injection. As shown in Figure 10, the device architecture was ITO/PEI-modified ZnO (50 nm)/CsPb(Br/I)_3_ (60 nm)/4,4′-bis(carbazole-9-yl)biphenyl (CBP)/4,4′,4″-tris(carbazol-9-yl)triphenylamine (TCTA) (50 nm)/MoO_x_/Au, in which the double HTL (CBP and TCTA) ensure enough hole injection. As a result, the PeLED gave an EQE up to 6.3%, which is the highest value reported among perovskite nanocrystal LEDs at that time. Since PEI is effective to enhance the electron injection and maintain charge neutrality of CsPbX_3_ emitters, Yu et al. then used a more efficient red emitter, PbS capped CsPbI_3_, in the similar device architecture of ITO/PEI-modified ZnO/CsPbI_3_/TCTA/MoO_x_/Au, exhibiting a peak EQE of 11.8% [53]. More recently, Yu et al. applied the architecture of Ag cathode/PEI-modified ZnO/CsPbI_3_/TCTA/MoO_x_/Au onto a photopolymer flexible substrate, attaining a flexible all-inorganic PeLED with the EQE of 8.2% [54].

### 3.5. Schemes to Increase the Charge Balance

Since EQE is directly decided by the charge balance, the increase of charge balance is essential to high-performance all-inorganic PeLEDs. In particular, excess electrons occur easily when organic ETLs are used because of the barrier-free electron injection behavior between organic ETLs and all-inorganic perovskite EMLs. To resolve this issue, the enhancement of hole injection is conducive [220,221,222,223,224,225]. Alternatively, the reduction of electron injection is also useful. This scheme is very flexible, since plenty of means can be utilized to reduce the electron injection, such as the insertion of an insulating layer between the ETL and EML [42], the use of electron transport materials with low mobilities [226], the employment of ETLs with thick thickness [227] and the introduction of HTL between the ETL and EML [228]. As these schemes are well reported in various kinds of LEDs, it is also possible to develop all-inorganic PeLEDs with such schemes.

**Figure 11:**
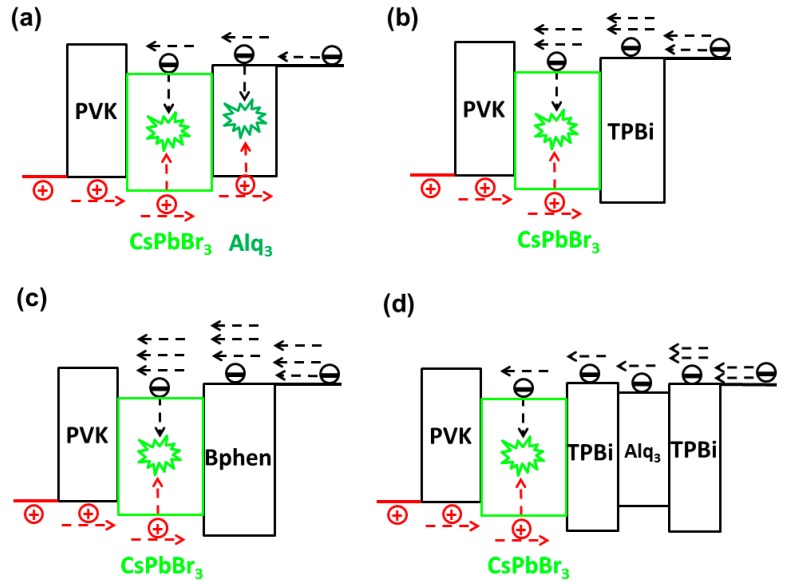
The working mechanisms of CsPbBr_3_ PeLEDs: (**a**) Device with Alq_3_ ETL, (**b**) device with TPBi ETL, (**c**) device G3 with Bphen ETL and (**d**) device with TPBi/Alq_3_/TPBi ETL. The red and black arrows represented the hole and electron transport, respectively. Reproduced from reference [229], with permission from John Wiley and Sons, 2018.

To balance the charges, Liu et al. reduced the electron injection by adopting a proper ETL in all-inorganic PeLEDs [229]. In detail, tris(8-hydroxyquinoline) aluminum (Alq_3_) was incorporated into TPBi to form an ETL TPBi/Alq_3_/TPBi, simultaneously enabling charge balance and confinement. The green PeLED with the device architecture of ITO/PEDOT:PSS (40 nm)/PVK (10 nm)/CsPbBr_3_ (20 nm, 10 mg mL^−1^, 2000 rpm, 45 s)/TPBi (15 nm)/Alq_3_ (10 nm)/TPBi (10 nm)/Cs_2_CO_3_ (1 nm)/Al (100 nm) exhibited a maximum EQE of 1.43%, which was 191%, higher than that of PeLEDs with conventional ETL TPBi (35 nm). The maximum current density of the PeLED with TPBi/Alq_3_/TPBi was 186 mA cm^−2^. The highest EQE was obtained at a very low current density of 0.44 mA cm^−2^, which is a common issue for all-inorganic PeLEDs [47]. As shown in Figure 11, since the electron mobility of Alq_3_ (1.4 × 10^−6^ cm^2^ V^−1^ s^−1^) was almost equal to the hole mobility of PVK (1.0 × 10^−6^ cm^2^ V^−1^ s^−1^) while the electron mobility of TPBi (3.3 × 10^−5^ cm^2^ V^−1^ s^−1^) and Bphen (3.9 × 10^−4^ cm^2^ V^−1^ s^−1^) was much higher than the hole mobility of PVK, the incorporation of Alq_3_ could impede the electron transport in order to balance the holes. In other words, the Alq_3_ reduced the number of electrons passing through the ETL. As a result, a relatively appropriate number of electrons could reach the CsPbBr_3_ EML. On the other hand, TPBi could confine the hole transport, further guaranteeing the performance. However, it is deserved to point out that some perovskite papers that discuss deliberate charge imbalance being okay, since the charge imbalance may result in good performance (e.g., ultralow voltages) [86].

### 3.6. Methods to Decrease the Charge Leakage

In some cases, holes and electrons can escape from the perovskite EMLs to the ETL and HTL, respectively. For example, holes may arrive at the ETL if there is no hole blocking layer or the energy level of ETLs is not deep enough compared with that of perovskite EMLs. On the other hand, electrons may reach the HTL when no electron blocking layer exists or the energy level of HTLs is not shallow enough. To loosen these bottlenecks, the addition of charge blocking layer or the charge transport layer with high charge blocking capability is required [230,231,232,233,234]. In addition, 3D perovskite film is prone to be rough or discontinuous because of the crystalline property, resulting in strong leakage current [235,236,237,238,239]. Thus, the performance of all-inorganic PeLEDs will be deteriorated due to the poor film morphology.

**Figure 12:**
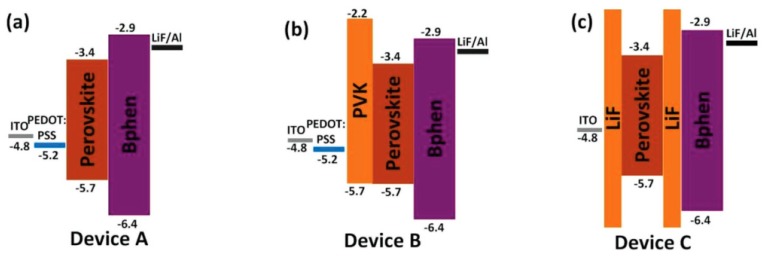
The energy level diagram of three types of PeLEDs. The active layers were PEDOT:PSS/perovskite/Bphen for Device A (**a**), PEDOT:PSS/PVK/perovskite/Bphen for Device B (**b**), and LiF/perovskite/LiF/Bphen for Device C (**c**). Reproduced from reference [240], with permission from John Wiley and Sons, 2018.

To form a dense and uniform perovskite film with full coverage, Wu et al. proposed an “insulator–perovskite–insulator” device architecture to tailor PeLEDs [240]. As shown in Figure 12, the architecture of Device C was ITO/LiF (4 nm)/perovskite/LiF (8 nm)/Bphen (60 nm)/LiF (0.8 nm)/Al (100 nm), where perovskite was sandwiched between the two LiF insulating layers. For various types of perovskites, the proposed device architecture could simultaneously induce charges into perovskite crystals, block current leakage via pinholes in the perovskite film and avoid exciton quenching. Consequently, the EQE of FAPbBr_3_ PeLEDs was increased to 5.53%, which was much higher than that of the control device with the conventional HIL (PEDOT:PSS, Device A) or PEDOT:PSS/PVK (0.174%, Device B). For the case of MAPbBr_3_ PeLEDs, the EQE was increased from 0.057% to 2.36%. In terms of the all-inorganic PeLEDs, both current efficiency and lifetime of CsPbBr_3_ PeLEDs were improved from 1.42 and 4 h to 9.86 cd A^−1^ and 96 h compared with the control device.

## 4. Summary and Outlook

Since all-inorganic PeLEDs can show high efficiency, low driving voltage, bright luminance, outstanding color-purity and long lifetime, the excellent characteristics have rendered that all-inorganic PeLEDs are very promising for the new-generation displays and lighting. Nowadays, the efficiency of state-of-the-art all-inorganic PeLEDs can be comparable to that of the best OLEDs and CdSe-based QD-LEDs. In this review, we have mainly focused on recent advances in the device engineering of all-inorganic PeLEDs. Particularly, we have emphasized the emergence of all-inorganic PeLEDs, strategies to improve the hole injection, approaches to enhance the electron injection, schemes to increase the charge balance and methods to decrease the charge leakage. The detailed performances for all-inorganic PeLEDs have been described in Table 1.

After approximate five years of development, the performance of all-inorganic PeLEDs has been step-by-step enhanced. In addition to the optimization of perovskite emitters, the PeLED performance has been greatly boosted by the innovation of device engineering. Currently, all-inorganic PeLEDs are also being explored in flexible as well as transparent optoelectronics, which further widen their general applications [241,242,243]. In addition, it can be easily predicted that all-inorganic PeLEDs will show higher performance by virtue of effective outcoupling technologies [244,245,246,247], despite negligible attention has been paid on the improvement of the outcoupling factor thus far. Moreover, compared with OLEDs and CdSe-based QD-LEDs, the outstanding color-purity of all-inorganic PeLEDs is more desirable for displays [248,249,250].

Nowadays, there are still many challenges hindering the real commercialization of all-inorganic PeLEDs, such as the efficiency, efficiency roll-off, toxicity and particularly for the operational stability. For the issue of efficiency, although the EQE of all-inorganic PeLEDs have been demonstrated to show >20%, the current efficiency and power efficiency are still not comparable to those of the best OLEDs and CdSe-based QD-LEDs [251,252,253]. Besides, the efficiency of blue all-inorganic PeLEDs is urgently needed to be enhanced, considering the highest EQE of blue device is only 1.9% [55]. To boost the efficiency, the charge injection, transport, balance and leakage should be carefully manipulated, which is also beneficial to the efficiency roll-off, color stability and lifetime. To solve the issue of toxicity, lead-free all-inorganic perovskites are helpful, although their performance is not satisfactory enough [245,246,247,248,249,250,251,252,253,254,255,256,257,258].

For the issue of lifetime, it is a key factor to determine whether all-inorganic PeLEDs can meet the demand of the commercial demands. In general, the lifetime of ≥10 000 h at ≥1000 cd m^−2^ is required for the commercial solid-state lighting products and ≥100 000 h at ≥100 cd m^−2^ is needed for the display applications [259,260,261]. Hence, there is still much room to prolong the lifetime. Previously, some effective approaches have been reported to enhance the stability of all-inorganic perovskite materials, including resistance to moisture and oxygen. For example, Zou et al. demonstrated a method to improve the formation energies of perovskite lattices by doping Mn^2+^ into CsPbX_3_ QDs, achieving stable CsPbX_3_ even at high temperatures up to 200 °C under ambient air conditions [262]. Ding et al. reported an approach to prepare stable and water-soluble CsPbX_3_/SiO_2_ nanocomposites by encapsulating the CsPbX_3_ QDs into silica nanoplates, where the application of CsPbX_3_/SiO_2_ in white LEDs and cell imaging showed ultrastability and high biocompatibility [263]. Zhu et al. provided a scheme to improve the stability of CsPbBr_3_ nanocrystals film by depositing Al_2_O_3_ on nanocrystals film surface for via plasma enhanced atomic layer deposition [264], where the dense Al_2_O_3_ film also exhibited an encapsulation effect [265]. Shi et al. presented an approach to combine localized surface plasmons and core/shell nanostructure configuration in a single PeLED, where the PeLEDs without encapsulation presented a substantially improved operation stability against water and oxygen degradation (30-day storage in air ambient, 85% humidity) [266]. Another effective approach is the hybridization of CsPbX_3_ with other matrix materials (e.g., synthesizing a chemically and structurally stable CsPbBr_3_ perovskite material by using polymer nanofiber as the protective layer [267], presenting a high molecular weight polymer matrix (polymethylmethacrylate, PMMA) to incorporate into CsPbBr_3_ QDs to improve its stability and maintain excellent optical properties [268]). Apart from the exploration of stable perovskite emitters, the further enhancement of device engineering is also urgently necessary (e.g., the use of stable inorganic charge transport layers, the exploitation of insulators, the introduction of advanced encapsulation techniques) [269,270,271,272,273,274]. After solving the mentioned issues, the prospect for mass production for all-inorganic PeLEDs will be bright and the proposed solutions are also beneficial to the related optoelectronic fields [275,276,277,278,279]. In particular, since the design concept of sensors [280,281,282] and photodetectors [283,284,285] are somewhat similar to that of LEDs, the new-emerging all-inorganic perovskite materials have also been extensively investigated for these applications.

## Figures and Tables

**Table 1:** Summarized performances for representative all-inorganic PeLEDs.

PeLEDs ^a^	V_on_^b^ (v)	EQE_max_^c^ (%)	PE_max_^d^ (lm W^−1^)	CE_max_^e^ (cd A^−1^)	L_max_^f^ (cd m^−2^)
Ref. [19]	4.2	0.12	0.18	0.43	946
Ref. [47]	2.6	8.73	31.7	26.2	1660
Ref. [49]	3.4	6.27	5.24	13.3	15185
Ref. [52]	1.9	6.3	4.05	3.4	2216
Ref. [72]	2.5	0.06	-	0.19	1337
Ref. [93]	3.0	0.008	-	0.035	407
Ref. [201]	-	2.8	-	12.3	20636
Ref. [212]	3.0	2.39	-	2.25	3809
Ref. [229]	4.8	1.43	1.84	4.69	452
Ref. [240]	-	2.99	-	9.86	~13000

^a^ Representative all-inorganic PeLEDs. ^b^ Turn-on voltage. ^c^ Peak EQE. ^d^ Peak PE. ^e^ Peak CE. ^f^ Peak luminance.
